# New tools for cell reprogramming and conversion: Possible applications to livestock

**DOI:** 10.21451/1984-3143-AR2019-0043

**Published:** 2019-10-23

**Authors:** Fulvio Gandolfi, Sharon Arcuri, Georgia Pennarossa, Tiziana A.L. Brevini

**Affiliations:** 1 Department of Agricultural and Environmental Sciences - Production, Landscape, Agroenergy, University of Milan, Italy.; 2 Department of Health, Animal Science and Food Safety, University of Milan, Italy.

**Keywords:** cell reprogramming, epigenetic erasing, mechanosensing

## Abstract

Somatic cell nuclear transfer and iPS are both forms of radical cell reprogramming able to transform a fully differentiated cell type into a totipotent or pluripotent cell. Both processes, however, are hampered by low efficiency and, in the case of iPS, the application to livestock species is uncertain.

Epigenetic manipulation has recently emerged as an efficient and robust alternative method for cell reprogramming. It is based upon the use of small molecules that are able to modify the levels of DNA methylation with 5-azacitidyne as one of the most widely used. Among a number of advantages, it includes the fact that it can be applied to domestic species including pig, dog and cat.

Treated cells undergo a widespread demethylation which is followed by a renewed methylation pattern induced by specific chemical stimuli that lead to the desired phenotype. A detailed study of the mechanisms of epigenetic manipulation revealed that cell plasticity is achieved through the combined action of a reduced DNA methyl transferase activity with an active demethylation driven by the TET protein family. Surprisingly the same combination of molecular processes leads to the transformation of fibroblasts into iPS and regulate the epigenetic changes that take place during early development and, hence, during reprogramming following SCNT.

Finally, it has recently emerged that mechanic stimuli in the form of a 3D cell rearrangement can significantly enhance the efficiency of epigenetic reprogramming as well as of maintenance of pluripotency. Interestingly these mechanic stimuli act on the same mechanisms both in epigenetic cell conversion with 5-Aza-CR and in iPS.

We suggest that the balanced combination of epigenetic erasing, 3D cell rearrangement and chemical induction can go a long way to obtain ad hoc cell types that can fully exploit the current exiting development brought by gene editing and animal cloning in livestock production.

## Introduction

After many decades of heated debate, the birth of Dolly proved, beyond any doubt, that each cell of our organism retains all the information originally present in the zygote. Somatic cell nuclear transfer is indeed the ultimate form of cell conversion since it enables the birth of a new individual from a single differentiated cell. Consequently, this also implies that differentiation does not permanently silence any of this information and that, providing the right stimuli, such information can be restored and become functional again.

A slightly less dramatic form of somatic cell reprogramming is achieved through the transfection of four master genes to obtain what are universally known as induced pluripotent stem (iPS) cells. In this case instead of a whole individual it is possible to generate several of its parts. As indicated by their name, iPS cells can potentially generate each of the approximately 200 cell types that constitute a human or an animal body. These properties are not exclusive of iPS but were first described in embryonic stem cells (ESC) that are derived from pre-implantation embryos. In both cases, cells are characterised by a stable pluripotency which is not found in any cell neither in the embryo nor in any other phase of life. It can rather be considered a cell culture artefact that captures a physiological stage naturally transient in early development.

As we summarised in a previous paper (Gandolfi *et al*., 2012), the ability to transform such transient stage into a permanent property is limited to a very small number of species (mostly mice and primates including humans) while it has proved very elusive in livestock species. The reason for such difference is still unclear. One hypothesis linked the problematic derivation of bona fide ESC in domestic species to the prolonged pre-implantation period typical of domestic animals. However, this looks unlikely because also the derivation of bona fide iPS cells has proved to be elusive in these species.

Since stable pluripotency does not exist in nature, living organisms utilise different mechanisms for the day to day replacement of worn or damaged cells. Differentiation is physiologically associated with cell proliferation and, on the contrary, fully differentiated cells lose their ability to proliferate. For this reason, maintaining tissue homeostasis is the functional task of the so-called somatic stem cells. These small groups of dedicated cells reside in well-defined niches which modulate their proliferative capacity in response to the functional requirements of the organism. Stem cells proliferate into intermediate, partially differentiated populations that cease to replicate once they become fully matured and ready to perform their specific function (Gandolfi and Brevini, 2018).

In some circumstances we know that such process can be reverted, and a fully differentiated cell may resume its proliferative ability and give rise to a different cell type in response to specific stimuli (Brevini and Gandolfi, 2013).

Understanding what stimulates and regulates the transformation of a differentiated cell into a different type of cell is a fascinating topic of study and being able to harness this process has a wide range of medical and commercial applications.

Aim of this short review is to provide the reader with an overall perspective of the most recent concepts about the relationships that exist between stem cells, cell conversion and cloning in livestock.

## Pluripotency *in vivo* and *in vitro*

In the early phases of mammalian embryonic development, three germ layers, the endoderm, mesoderm and ectoderm are formed; each one gives rise to a different set of tissue types and contributes to specific organs. Stem cells are classified according to their potency that can span from unipotency when only a single cell type can be generated, to multipotency, when a stem cell can originate to all or many cells of a single germ layer.

When a stem cell can differentiate into cells that arise from all three germ layers, is defined as pluripotent. In nature, pluripotency is limited to the epiblast, a transient tissue that exists only for a brief stage period of embryonic development, before giving origin to the three germ layers. Therefore, the epiblast is not a kind of stem cell because it lacks the property of asymmetric division and stable pluripotent cells are not a physiological component of the body but are created only *in vitro* (Smith, 2001).

*In vitro*, it has been possible to transform pluripotency from a transient state into a permanent property of stable cell lines. These can be derived directly from early embryos generating the embryonic stem cells (ESC) or can be obtained through the transfection of 4 transcription factors into somatic cells, generating the induced pluripotent stem cells (iPS). Both kind of pluripotent stem cells can be readily derived in mouse and human but it has proved much more challenging if not outright impossible, to derive pluripotent cells in livestock species, as detailed in some recent reviews (Brevini *et al*., 2010; Koh and Piedrahita, 2014; Kumar *et al*., 2015; Soto and Ross, 2016). Pluripotent cell lines in these species are defined as ES-like since they show several major deficiencies, ranging from a short life in culture to the lack of controlled pluripotency or of the ability to form chimeras (Talbot and Blomberg le, 2008). Despite the extensive research activity, it is still unclear why it is not possible to derive truly pluripotent ESC or iPS from these species.

Since ESC originate from the epiblast the question arises if the lack of domestic animals ESC is due to the lack of appropriate culture conditions or the epiblast from these species is inherently different so that “suspending” it’s properties *in vitro* may not be possible.

The process of epiblast formation in mouse is known in great detail (Rossant and Tam, 2009). During the first embryonic divisions, all blastomeres are totipotent and all express the transcription factor Octamer Binding Protein 4 (OCT4). The first differentiation process consists in the generation of trophectoderm (TE) and inner cell mass (ICM) cells from their unique totipotent blastomere precursors. This is marked by the restriction of OCT4 expression to ICM cells, which is caused by its repression by caudal type homeobox 2 (CDX2). The result is that TE cells express CDX2 and ICM cells express OCT4. ICM cells will then undergo a further differentiation leading to the formation of the hypoblast, that will lose OCT4 expression, and of the epiblast that will retain it.

Mouse epiblast differentiation and restriction of Oct4 expression to this tissue is completed by E3.5. By E5.5 mouse embryos are embedded into the uterine wall. Human embryos go through the same changes but at a slower pace with OCT4 restriction to the epiblast completed by E6 and implantation taking place at E7-9 (Rossant, 2015).

When we examined the distribution of OCT4 in bovine embryos we soon realized that it is not as tightly restricted to ICM as described in mouse and human embryos but it was ubiquitously expressed also in expanded blastocysts (Van Eijk *et al*., 1999). When observations were extended to later stage embryos it was determined that OCT4 restriction to the epiblast is completed only by E11 in bovine (Berg *et al*., 2011) and E8-9 in pig (Hall *et al*., 2009) embryos.

Based on this different timing, attempts have been performed using day 10-12,5 elongated pig blastocysts, using the knowledge that late, or so-called “primed”, epiblast responds better to FGF2 than to LIF (Alberio *et al*., 2010). Indeed, results were encouraging with cell lines showing a robust self-renewal and the ability to differentiate into precursor cells derived from all three germ layers as well as into trophectoderm and germ cell precursors. However it is possible to obtain similar results with day 6 blastocysts using both LIF and FGF2 (Brevini *et al*., 2010).

At present, culture conditions are still far from being elucidated. Telugu *et al*. (2011) derived LIF-dependent, so-called naive, pluripotent stem cells from the ICM of porcine blastocysts by up-regulating expression of KLF4 and POU5F1 with lentivirus vector. Haraguchi *et al*., (2012) generated porcine ES-like cells from the ICM of porcine embryos by using inhibitors, CH99021 and PD184352. Recent results showed that the combination of bFGF, EGF, Activin-a, ITS, and KO Serum is also effective to promote attachment, outgrowth and expansion of porcine ICMs and generate ESC-like cells (Hou *et al*., 2016).

Given the possibility that the specific morphological and functional characteristic of domestic ungulate pre-implantation embryos may have a profound influence on the possibility to derive ESC lines, it was interesting to see whether the forced induction of pluripotency achieved with the iPS technology made it possible to obtain ungulates bona fide pluripotent stem cells bypassing the embryo as a starting material.

Indeed iPS have been obtained in wide range domestic ungulates, but in some instances, expression of the exogenous pluripotency genes was not down regulated or was artificially maintained (Gandolfi *et al*., 2012). In the first case, this made it difficult to induce teratoma formation. In the latter, the absence of expression induced a rapid differentiation in pig (Esteban *et al*., 2009; Wu *et al*., 2009), sheep (Li *et al*., 2011) and cow (Sumer *et al*., 2011) cell lines. More importantly, the ability of livestock iPSCs to generate chimeras was very low and even lower was their ability to contribute to the germ line (West *et al*., 2011). The results are consistent with the fact that most of these cell lines show the characteristics of the primed type.

The recent developments of new media were able to convert pig primed cell lines into the naïve type and to confer higher clonal properties to primed lines renewing our hopes that further developments may be achieved in livestock species able to generate a chimera in the near future (Ma *et al*., 2018).

At present, however, available data suggest that true LIF-dependent naïve/ESC equivalent to those of mouse cannot be obtained in ungulates, possibly due to some inherent characteristic of their epiblast.

Whether or not in the future will be worthwhile to pursue this line of research in domestic or in other species is open to debate. A large body of evidence shows that the differentiation of pluripotent stem cells, both embryonic or induced, is difficult to control and it is dangerously similar to neoplastic transformation. On the contrary new approaches have been developed that enable the study of the differentiation process and, at the same time, look much safer for clinical applications, as described in the next section.

## Epigenetic cell conversion

Following on from the pioneering work of Taylor and Jones (1979), many groups have reported that it is possible to use small molecules and epigenetic modifiers in order to directly convert an adult cell into an alternative differentiated cell type (Brevini *et al*., 2014; Chandrakanthan *et al*., 2016; Manzoni *et al*., 2016; Pennarossa *et al*., 2013). Several protocols using epigenetic modifiers have been developed that can push cells to a transient ‘less committed state’, increasing cell plasticity for a short time, sufficient to redirect them towards a different cell type (Brevini *et al*., 2014; Chandrakanthan *et al*., 2016; Harris *et al*., 2011; Mirakhori *et al*., 2015; Pennarossa *et al*., 2014, 2013). The general concept of these experiments is that DNA methylation plays a fundamental role during cell differentiation during early embryonic development and cell lineage specification. For this reason, 5-azacytidine (5-aza-CR), a well-characterised DNA methyltransferase inhibitor, has often been used to remove the epigenetic ‘blocks’ that are responsible for tissue specification (Brevini *et al*., 2014; Chandrakanthan *et al*., 2016; Pennarossa *et al*., 2013). Because of its powerful effects, 5-aza-CR induces global DNA hypomethylation (Christman, 2002) and gene reactivation (Jones, 1985) facilitating somatic cells switching from one phenotype to another (Glover *et al*., 1986; Harris *et al*., 2011; Taylor and Jones, 1979). A brief exposure to 5-aza-CR can convert adult skin fibroblasts and granulosa cells into different cell types (Brevini *et al*., 2016, 2014; Pennarossa *et al*., 2017, 2014, 2013). Such fate switch is not limited to cells belonging to the same embryonic layer but can also occur between cells belonging to different embryonic layers.

After a 18 h-exposure to 5-aza-CR, cells acquire a ‘highly permissive state’ with significant changes in their phenotype and gene expression pattern accompanied by a decrease in global DNA methylation. Most surprisingly following exposure to this demethylating agent, cells acquire the morphological features distinctive of ESCs, iPSCs and pluripotent cells described by Tamada *et al*. (2006). These include reduced dimensions with large nuclei, global chromatin decondensation, as well as expression of pluripotency-related genes such as OCT4, NANOG, ZFP42 zinc finger protein (REX1) and SRY (sex determining region Y)-box 2 (SOX2). This was achieved not only with human and mouse but also with pig and dog fibroblasts (Brevini *et al*., 2016, 2014; Pennarossa *et al*., 2017, 2014, 2013) and the high efficiency and robustness of the process makes it the best option for working in domestic species (Gandolfi and Brevini, 2018).

**Figure 1:**
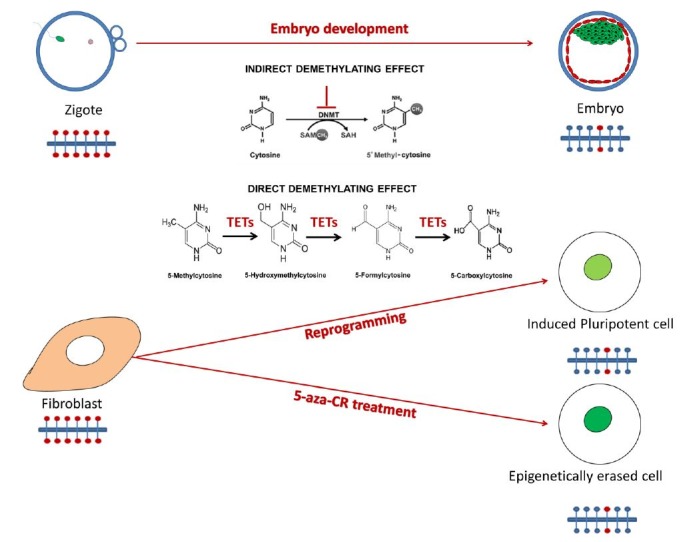
Inhibition of DNA methyl transferase (DNMT) enzymes combined with the activation of ten eleven translocation (TET) enzymes are at work in pluripotent and highly plastic cells. This indicates that cell plasticity is achieved and maintained through a common mechanism.

The mechanisms at work during epigenetic reprogramming are very similar to, or even the same, that regulate early embryonic development and the transformation of a somatic cell into an iPS (Fig.1). Pluripotent cells, either ESC or iPS, show a global cytosine demethylation (Leitch *et al*., 2013) which is crucial for maintaining the naive state and antagonising the self-activating differentiation signal, resetting the epigenome and re-establishing the pluripotency network (Grabole *et al*., 2013). In addition, downregulation of DNA methyl transferase enzymes (DNMT) is correlated with boosting symmetry in cell division (Jasnos *et al*., 2013), further supporting the idea that demethylation plays a major role in promoting self-renewal and maintaining cells in their most naive state. In agreement with these observations, cell fate restriction and subsequent differentiation is accompanied by a progressive build-up of DNA methylation. Indeed, it has been demonstrated that lineage specification is supported by dynamic epigenetic changes and genome-wide redistribution of DNA methylation that silence pluripotency genes and establish a phenotype-specific methylation pattern (Berdasco and Esteller, 2011; Oda *et al*., 2013). During cell fate commitment, pluripotency genes such as octamer-binding transcription factor 4 (Oct4) and Nanog undergo silencing and de novo DNA methylation in their promoter and enhancer regions. This hypermethylated state is then maintained in differentiated somatic cells (Epsztejn-Litman *et al*., 2008; Li *et al*., 2007).

As described above, cell phenotype can be reversed by transferring a somatic cell nucleus into an enucleated oocyte and, similarly, somatic cells transfected with specific reprogramming factors are converted into iPSCs. On the other hand, the identity of a differentiated cell is guaranteed by a unique methylation profile that maintains its lineage definition and prevents free transition among different cell types. Therefore, methylation must be removed in order to allow a switch in phenotype. For example, demethylation of pluripotency genes is a hallmark of somatic cell reprogramming into a pluripotent state (Gurdon and Melton, 2008; Takahashi and Yamanaka, 2006). Recently, studies have shown that experimental reprogramming requires active demethylation by the TET (ten-eleven-translocation) family of enzymes, which recently were identified to catalyse the conversion of cytosine-5 methylation to 5-hydroxymethyl-cytosine, an intermediate form potentially involved in demethylation (Mohr *et al*., 2011), leading to activation of epigenetically silenced pluripotency genes. In agreement with these observations, it has been reported that oocyte TET enzymes exhibited reprogramming activity for pluripotency gene reactivation during early embryonic development, after nuclear transfer and natural fertilisation (Gu *et al*., 2011). Together, these findings point to the possibility that TET enzymes play a key role in cell reprogramming as well as in mesenchymal to epithelial transition (MET) that characterise iPS formation. This hypothesis finds further support in experiments performed in mouse embryonic fibroblasts (MEFs), in which TET genes were inactivated, resulting in cell failure to undergo MET and a complete block of their reprogramming potential (Hu *et al*., 2014). These observations indicate that TET enzymes are indispensable for factor-driven reprogramming of somatic cells to iPSCs. Interestingly, the same authors showed that TET-deficient MEFs failed to reactivate microRNAs, such as miR-200 s, miR-200a and miR-200b, which play a critical role in MET and are upregulated in cells undergoing reprogramming. Indeed, Hu *et al*., (2014) showed that the expression of the miR-200 family diminished in TET-deficient MEFs, and this was accompanied by the reprogramming block. However, ectopic expression of miR-200s was able to restore the MET process and rescue up to 80% of the reprogramming efficiency of wild-type fibroblasts (Hu *et al*., 2014).

DNA methylation has also been shown to promote the adequate and proper regulation of gene expression, ensuring both temporal activation and spatial restriction, allowing cells to acquire distinct differentiation traits, stabilising the terminal cell phenotype and maintaining the established patterns by copying them onto daughter DNA strands during cell replication and division (Oda *et al*., 2013). Consistently, studies performed recently using culture media supplemented with two small kinase inhibitor (PD0325901 and CHIR99021) report the derivation of ESC lines with a level of hypomethylation higher than those derived using conventional media (Habibi *et al*., 2013; Leitch *et al*., 2013). In particular, also in this case, TET enzymes are involved since the two inhibitors increases their activity, boosting TET-mediated conversion of 5-mC to 5-hmC, which synergise with the simultaneous DNMT-related passive effect, easing cells into a ‘naive state’ in which the genome becomes hypomethylated and reminiscent of early blastomeres seen *in vivo* (Hu *et al*., 2014).

Epigenetic reprogramming can unleash the full differentiation potential of any cell type with none of the limits that constrain embryonic or induced pluripotent stem cells. It represents a step forward since it works through natural pathways instead of inducing artificial states. Therefore, in the near future we are likely to see a rapid expansion of this approach both in basic and in clinical research.

## Cell spatial arrangement in a 3D microenvironment

From all of the above we learned that manipulation of the epigenetic status of a somatic cell enables the quick and substantial increase of its plasticity that can be readily exploited for changing its fate and remodelling it according to our wishes. This is a very efficient and safe process because the raise of cell plasticity is temporary and reversible avoiding the danger linked to a permanent pluripotent state that severely limit the possible clinical use of iPS and ESC (Brevini *et al*., 2018).

However, in some circumstances the availability of more stable pluripotent cells may be of interest. One of such cases could be the use of pluripotent cells as nuclear donors for improving the currently low efficiency of somatic cell nuclear transfer.

As described in detail in a recent review numerous attempts have been performed to use epigenetic modifiers to improve SCNT efficiency (Curcio *et al*., 2017). However, at present, the possibility to significantly improve offspring production is controversial at best.

In this context, we studied the possibility to stabilize the high plasticity status obtained *in vitro* by the epigenetic reprogramming through the addition of a 3D microenvironment. In particular we used polytetrafluoroethylene (PTFE) micro-bioreactors to induce cells to self-assemble and form multicellular spheroids, displaying a uniform size geometry (Pennarossa *et al*., 2019). This stems from previous studies indicating that PTFE is able to efficiently encourage cell aggregation, facilitating the formation of embryoid bodies from murine ESC (Sarvi *et al*., 2013) or the establishment of olfactory ensheathing cell spheroid structures (Vadivelu *et al*., 2015). Our results demonstrate the 3D cell rearrangement, obtained within the microbioreactors induced global DNA demethylation and elevated transcription of pluripotency markers. Ultrastructural analysis demonstrated that cells in the 3D spherical structures showed significant intercellular spaces, high nucleus to cytoplasm ratio, nuclei containing euchromatin and large reticulated nucleoli. Cytoplasm was characterized by the presence of free ribosomes, polyribosomes, elongated tubular mitochondria, well-developed rough endoplasmic reticulum, Golgi complexes, few reticulum cisternae and lipid droplets. All these features resemble the morphology typical of undifferentiated cells like ESC and iPS, and remind of the inner cell mass (ICM) of blastocysts (Courtot *et al*., 2014; Efroni *et al*., 2008; Lai *et al*., 2015; Liang and Zhang, 2013; Meshorer *et al*., 2006; Meshorer and Misteli, 2006; Sathananthan *et al*., 2002). These observations suggest that the use of PTFE microbioreactors encourages cell aggregation and boosts the induction and stable maintenance of morphological properties typical of pluripotent cells.

Molecular analysis showed that PTFE encapsulated cells remained significantly hypomethylated for the entire length of the experiments. Furthermore, our results showed that epigenetic erasing led to an increased expression of the ten-eleven translocation family member TET2, accompanied by the onset of the pluripotency-related genes, OCT4, NANOG, REX1 and SOX2, as well as the up-regulation of EPCAM, and CDH1 genes, confirming and expanding previous studies carried out in our laboratory (Brevini *et al*., 2014; Manzoni *et al*., 2016; Pennarossa *et al*., 2014, 2013). As we described above, this is the same mechanism taking place in epigenetic reprogramming which, in turn, replicates the methylation changes taking place during iPS reprogramming with the combined effect of reduced DNMT activity with the active demethylation controlled by TET proteins (Hysolli *et al*., 2016). The two play an essential role in pluripotency maintenance and the acquisition of a high plasticity phenotype (Ito *et al*., 2010; Tahiliani *et al*., 2009), resulting in the decrease of fibroblast-specific marker (THY1), the onset of pluripotency-related genes (OCT4, NANOG, REX1, and SOX2), and the upregulation of key MET markers (EPCAM, CDH1).

**Figure 2:**
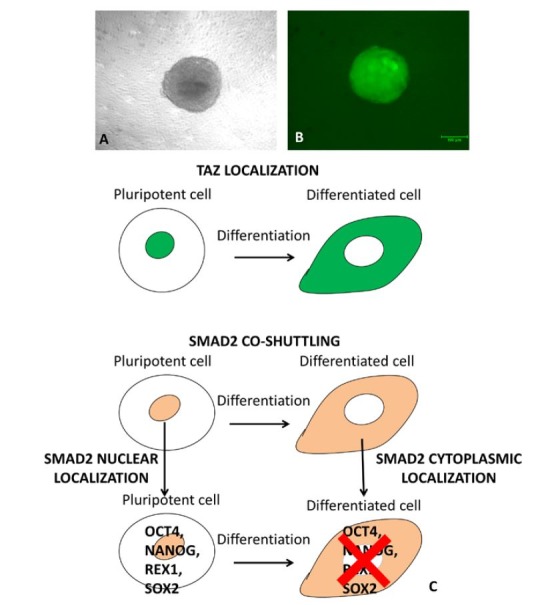
When cells are allowed to grow in a 3D arrangement, obtained within PTFE microbioreactors (panel A), DNA undergoes a global demethylation and transcription of pluripotency markers increases (e.g. Oct4 as shown in panel B). This is achieved by the nuclear translocation of TAZ protein that drives SMAD2 into the nucleus that, in turn, activates the transcription of Oct4, Nanog, Rex1 and Sox2.

Interestingly, these changes were promoted and stably maintained by the use of the PTFE microbioreactor, suggesting that 3D cell confinement boosts pluripotency gene transcription and maintains long-term cell plasticity. These morphological and molecular changes were accompanied by the activation of the Hippo-signalling pathway with distinctive modifications in the transcriptional cofactor TAZ localization. In particular, the 3D cell confinement encouraged TAZ nuclear retention, that was stably maintained for the entire length of the experiments. TAZ localization was mirrored by a parallel nuclear accumulation of signal transducer SMAD2 (Fig.2). This evidence is in line with previous reports that indicate a direct interaction between TAZ and SMAD proteins, where TAZ defines a hierarchical system, regulating SMAD complexes shuttling and coupling to the transcriptional machinery (Ohgushi *et al*., 2015; Varelas *et al*., 2008). These observations are even more intriguing, given the fact that the Hippo signalling pathway and its activators are highly expressed in the mammalian embryo and have been recently shown to contribute to and to improve early embryonic development (Yu *et al*., 2016). Mechanosensing-related activation of such pathway is therefore likely to enhance epigenetic reprogramming and plasticity.

## Conclusions

Careful modulation of the epigenetic make-up provides an efficient and safe way to change the state of any somatic cells. The increased plasticity is reversible and transient, making it much more physiological than the permanent pluripotency of ESC and iPS.

Recent developments revealed a surprising overlap among the molecular mechanisms that control cell reprogramming, even if it is achieved through different techniques, and the regulatory pathways acting in the early embryo.

**Figure 3:**
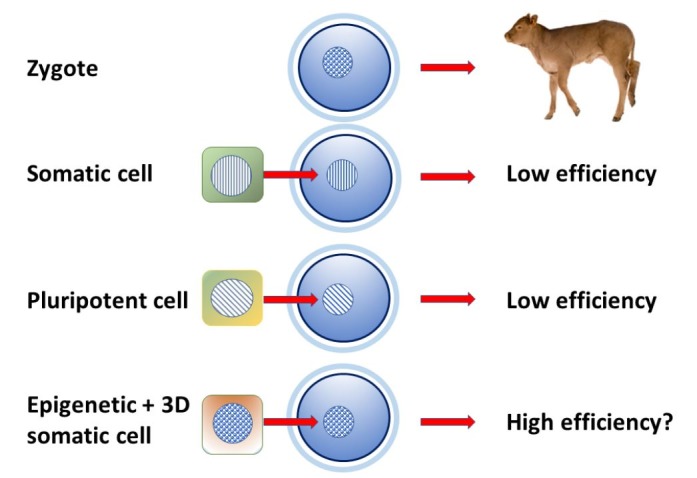
Efficiency of somatic cell nuclear transfer has remained low though the years. It is thought that the different epigenetic status between the donor cells and the zygote nucleus may be the main reason. The use of ESC of iPS pluripotent cells has brought only small improvements if any. We hypothesise that the understanding of the molecular mechanisms common to different reprogramming methods may lead to an accurate control of the nuclear epigenetic status that will resemble that of the zygote, thereby significantly increasing SCNT efficiency and unleashing the full potential of genome editing in livestock species.

In the near future it will be interesting to see if it is possible to harness the full potential of these mechanisms to achieve an accurate epigenetic resetting. For instance, we know that the low efficiency of SCNT is largely due to the short time available for the nucleus to undergo the extensive epigenetic reprogramming that takes place after fertilization. Even the use of ESC or iPS has been unable to significantly improve it, possibly because pluripotent cells are more similar to the epiblast than to the zygotic nucleus. We can hypothesise that the balanced combination of epigenetic erasing, 3D cell rearrangement and chemical induction can transform the epigenetic status of the somatic nucleus into that found in the zygote, in practice, reprogramming it before its transfer, so that it can follow the physiological evolution leading to the complete development (Fig. 3). This may prove to be a novel tool to obtain cell nuclei much more amenable to a correct reprogramming within the short time frame provided by SCNT. The resulting improvement may enable to fully exploit the exiting developments promised by gene editing in livestock production.
